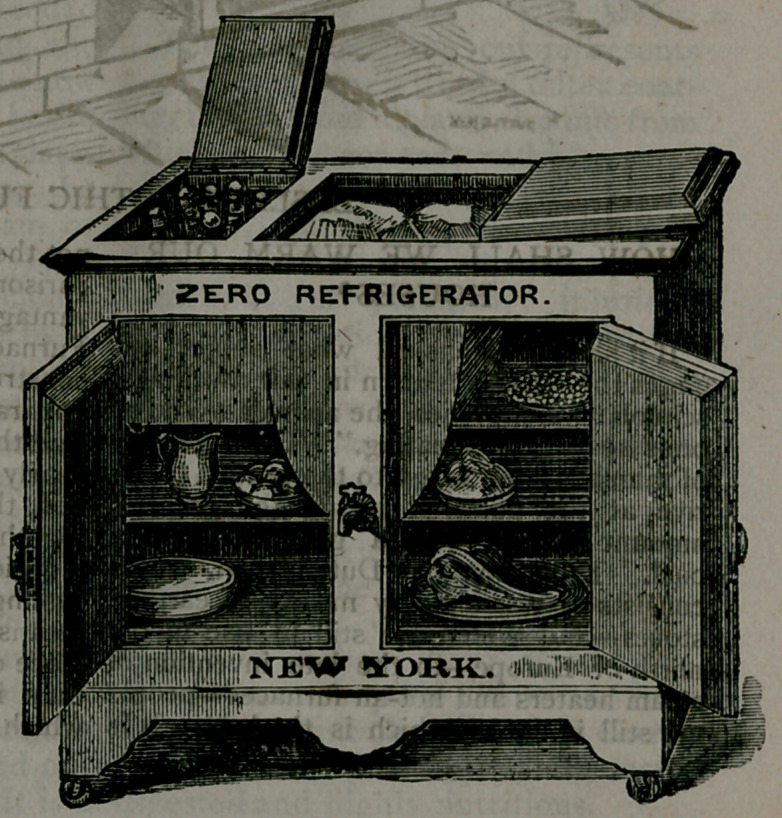# Zero Refrigerator

**Published:** 1875-09

**Authors:** 


### THE ZERO REFRIGERATOR,

Represented by the accompanying cut, we believe to be the best meat, fruit and ice preserver in the world.

The qualities which give it this pre-eminence are described by a cotemporary as follows:

"Why has the water from the melted ice in an ordinary refrigerator a bad taste? Simply because all the odors and flavors, good and bad, of the materials cooled in the ice-box, and also the vapors with which the atmosphere entering the box is charged, condense on the surface of the ice, and mingle with the water proceeding from it. In order to be convinced of the fact that impurities of the atmosphere are condensed on the surface of ice, put one large lump of ice on an open dry dish and place it in a crowded room, especially when lights are burning, say at an evening party, (a dancing party is still better,) and after twenty or thirty minutes drink the water which has collected from the melting ice on the bottom of the dish, and you will find it to be of an abominable taste. No wonder; it contains the products of the perspiration, of the breath, all the smells of the people in the room, and that of the gas or kerosene besides. Therefore, if you want pure ice-water, put your ice in a pitcher closed with a cover, where the air in the room cannot reach it, and let it melt beyond contact with the air currents which will be generated by the cooling effect of the ice, which causes the air to contract, become heavier, and continually to descend around the ice, and deliver its aroma to the ice-water.

In connection with the above, we call attention to Lesley's (of 226 West Twenty-third street, New York) Zero Refrigerator, illustrated in the accompanying engraving, and in which the ice is contained in a separate metallic vessel, against the very cold sides of which the vapors and odors (of the meat, fish, vegetables, etc., preserved in the box) are condensed and discharged below by a separate tube, which delivers a very unpalatable liquid, while the pure water proceeding from the ice melting in the closed box, may be drawn by a separate faucet, seen in the middle of the front, and procure plenty of fresh ice-water, instead of being lost, as is the case in ordinary ice-boxes or so-called refrigerators.

There is incidental economy connected with this arrangement; firstly, the retention of the water proceeding from the melting ice, which, having the low temperature of 32 degrees, may still be of some service in cooling by retaining it, in place of allowing it to run out and so wasting it at once; secondly, the surface of the metallic box, acting on the interior of the refrigerator as the cooling agency, is much larger and therefore more effective than the surface of the piece of ice, which in ordinary boxes constitute the only active cooling surface. It is not surprising, therefore, that all who have once used this style of refrigerator will never desire another kind."

---

Save your suds for the garden and plants, or to harden yards when sandy.

## Figures and Tables

**Figure f1:**